# Mechanisms for enhancement of *μ*-opioid receptor-mediated antinociception in the central nervous system through gallein-dependent modulation of downstream G protein *βγ* subunit signaling

**DOI:** 10.1016/j.molpha.2026.100123

**Published:** 2026-04-20

**Authors:** Jacob A. Ormes, Alan V. Smrcka, Emily M. Jutkiewicz

**Affiliations:** Department of Pharmacology, University of Michigan, Ann Arbor, Michigan

**Keywords:** Antinociception, Gallein, G protein-coupled receptor kinase, *μ*-Opioid receptor, Phospholipase C

## Abstract

The clinical utility of *μ*-opioid receptor (MOR) agonists is limited by side effects and tolerance. G protein *βγ* subunits (G*βγ*) are critical mediators of MOR signaling that not only drive anticonception but also regulate phospholipase C and G protein-coupled receptor kinase pathways that feedback and inhibit MOR signaling. Modulation of G*βγ* signaling with small molecules to preferentially inhibit feedback inhibition of MOR has been proposed as a strategy to enhance analgesia without exacerbating adverse effects. In this study, we investigated mechanisms by which gallein, a small-molecule G*βγ* signaling modulator, enhances MOR-mediated antinociception in vivo. Gallein pretreatment significantly enhanced the antinociceptive effects of different MOR agonists and nonopioid analgesics that act through Gi-coupled G protein-coupled receptors, with the strongest effects observed upon coadministration with morphine. Gallein-mediated enhancement of morphine-induced antinociception was abolished by the opioid antagonist naloxone and was absent in MOR knockout mice. Notably, systemic naloxone methiodide, which cannot cross the blood-brain barrier, did not prevent gallein-mediated enhancement of morphine-induced antinociception. Intracerebroventricular, but not intrathecal, administration of gallein enhanced morphine-induced antinociception. Gallein was also effective at enhancing the action of morphine in a hot plate assay, implicating supraspinal MOR dependency. Lastly, 10 S/T-A mice expressing phosphosite-deficient MORs lost responsiveness to gallein treatment, supporting a mechanism of action in which gallein inhibits G*βγ*-dependent inhibitory feedback phosphorylation of MOR in the central nervous system.

**Significance Statement:**

These findings provide insight into the mechanisms for potentiation of the antinociceptive effects of *μ*-opioid receptor (MOR) agonists via modulation of G protein *βγ* (G*βγ*) signaling pathways. The study demonstrates dependence on central MORs and requires the ability of MOR to be feedback-regulated through phosphorylation. Pharmacological modulation of G*βγ* signaling could serve as effective adjuncts to traditional opioid therapy.

## 1 Introduction

Considering both medical costs and lost economic productivity, a 2021 study estimated the economic costs of chronic pain in the United States at $722.8 billion.^1^ Although opioids are indeed effective for pain management,^2^ they come with unwanted side effects such as respiratory depression and constipation, abuse liability, and tolerance development. Much work has been done in the field of opioid research and G protein-coupled receptor (GPCR) physiology to attempt creating new opioid analgesics that circumvent these issues. The fact that these efforts have been met with limited success highlights a critical need for the development of new treatment strategies to improve the safety and efficacy of opioid analgesics.

Opioids primarily mediate their analgesic effects through the *μ*-opioid receptor (MOR). MORs are GPCRs that couple to the Gi family of G protein subunits. MORs regulate neuronal physiology largely via free G protein *βγ* subunits (G*βγ*) released upon activation and dissociation of the Gi *α*/*βγ* heterotrimer, and subsequent direct downstream effector regulation.^3^, ^4^, ^5^ Important G*βγ*-regulated effectors include inwardly rectifying potassium channels,^6^^,^^7^ N-type voltage-gated calcium channels,^8^ and soluble N-ethylmaleimide-sensitive factor attachment receptors,^5^^,^^9^ which inhibit neuronal activity, mediating antinociception. Opposing MOR stimulation of antinociception are feedback inhibition pathways, at least 2 of which are stimulated by G*βγ*. G*βγ* subunits are required for activation of GPCR kinase 2/3 (GRK2/3),^10^^,^^11^ which phosphorylate MORs at the carboxyl terminus, resulting in *β*-arrestin recruitment and desensitization.^12^^,^^13^ G*βγ* subunits also synergize with G*α*_q_ to activate phospholipase C (PLC)*β*3.^14^, ^15^, ^16^, ^17^ Previous work demonstrated that the potency of morphine in antinociception is enhanced in PLC*β*3^-/-^ mice,^18^ supporting the idea that MOR antinociceptive signaling is opposed by feedback inhibition by PLC activation, possibly through diacylglycerol-dependent protein kinase C (PKC) activation and MOR phosphorylation.

Two highly related small molecules, M119 and gallein, bind directly and reversibly to G*βγ* in vitro.^19^, ^20^, ^21^ Binding of either of these molecules inhibits interactions between G*βγ* and a subset of downstream targets including PLC*β* and GRK2,^19^^,^^22^, ^23^, ^24^, ^25^, ^26^ while leaving ERK, G*βγ*-regulated inwardly rectifying potassium channel, and N-type voltage-gated calcium channel activation unaffected.^19^^,^^27^^,^^28^ This effector selectivity is thought to be driven by M119/gallein binding to a subsite on G*βγ* that allows competition for only a subset of effectors, because each effector engages G*βγ*, using distinct protein contacts.^19^^,^^27^, ^28^, ^29^

Because M119/gallein blocks 2 pathways involved in inhibition of MOR-dependent antinociception, and because MOR signals largely through G*βγ* subunits released from Gi, it was hypothesized that M119/gallein might enhance MOR-dependent antinociception. Indeed, it was shown that M119 administered intracerebroventricularly (i.c.v.) enhanced the efficacy of morphine in a warm water tail withdrawal (WWTW) assay of antinociception.^19^^,^^22^ This enhancement was blocked in PLC*β*3^-/-^ mice, supporting the idea that M119-dependent blockade of G*βγ*-PLC*β*3 interactions mediates these effects.^19^ A subsequent study showed that systemic intraperitoneal (i.p.) administration of gallein enhanced the antinociceptive effects of morphine without altering MOR-mediated side effects (ie, constipation, reward, and respiratory depression).^30^ Multiple subsequent studies have confirmed and extended these initial findings.^14^^,^^31^^,^^32^ Measurements of enhanced postsynaptic currents in mouse brain slices from the periaqueductal gray demonstrated that gallein increases the potency of DAMGO-dependent inhibition of GABA release and that this effect was eliminated in slices from PLC*β*3^-/-^ mice.^14^ These data provide in vivo and ex vivo support for the idea that these compounds block G*βγ* interactions with effectors such as PLC*β*3 and GRK2, preventing feedback inhibition of MOR signaling, increasing morphine antinociceptive potency.

In this study, we further investigated in vivo mechanisms for gallein action and provide further support for these proposed mechanisms and determined whether systemic administration of gallein enhances MOR-dependent antinociception through actions in the central nervous system (CNS) or the periphery. We found that enhancement of morphine-induced antinociception by systemically administered gallein is dependent on central MORs, and these effects are generalizable across various MOR agonists, nonopioid Gi-coupled GPCR-based analgesics, and various nociceptive stimuli. Gallein had no effect on antinociception mediated by epibatidine, a non-GPCR--dependent agonist. Importantly, gallein was unable to shift a dose-response curve for the antinociceptive effects of morphine in mice lacking GRK and PKC phosphorylation sites in the C-terminus of MOR. Together these data provide further evidence for the proposed in vivo mechanism of gallein action in which gallein-dependent blockade of signaling pathways mediates feedback inhibition of MORs via binding to G*βγ* subunits released by Gi heterotrimers and that this requires engagement of MORs in the CNS. In addition, these data, in conjunction with previous reports, provide further evidence for a lack of generalized adverse effects of this approach, impacting primarily Gi-mediated antinociceptive signaling.

## 2 Materials and methods

### 2.1 Drugs and Reagents

The following drugs were used for the experiments: buprenorphine (Sigma-Aldrich), CP55940 (Sigma-Aldrich), clonidine (Cayman Chemical), epibatidine (Cayman Chemical), fentanyl (Sigma-Aldrich), gallein (Cayman Chemical), morphine (Henry Schein), nalbuphine (Sigma-Aldrich), naloxone (NLX) (Sigma-Aldrich), and naloxone methiodide (NLXM) (Sigma-Aldrich). All drugs were dissolved in 0.9% sterile saline except CP55940 and gallein. CP55940 was dissolved in a vehicle consisting of 5% EtOH, 5% castor oil, and 90% sterile saline and then further diluted in sterile saline for experiments. For i.p. systemic and intrathecal (i.t.) administration, gallein was dissolved in a vehicle consisting of 5% DMSO, 10% castor oil, and 85% sterile water. I.c.v. gallein treatments were dissolved in DMSO.

### 2.2 Animals

All animal procedures were conducted at the University of Michigan according to the National Institutes of Health Guide for the Care and Use of Laboratory Animals and with approval of the Institutional Animal Care and Use Committee. Wild-type (WT) C57BL/6 mice were purchased from Envigo and bred in-house for these studies. MOR^-/-^ mice were purchased from Jackson Laboratory and bred on a C57BL/6 background. Ten S/T-A mice were obtained from Stefan Schulz’s laboratory and bred on a C57BL/6 background. Mice were housed with a maximum of 5 animals per cage in clear polypropylene cages. Corn cob bedding and nestlets were provided for enrichment. Animals were housed in pathogen-free rooms maintained between 68 °F and 72 °F and 30% and 70% humidity. Rooms used a 12-hour light/dark cycle (lights on at 7 am and lights off at 7 pm) with free access to food. Experiments were conducted in the animals’ housing room during the light cycle. All mice used in experiments were between 7 and 12 weeks of age and weighed 16 to 28 grams. A combination of male and female mice was used for all experiments. Eight to sixteen animals per group were used for all experiments.

### 2.3 Pretreatment

Systemic gallein was administered i.p. 24 hours before morphine. I.c.v. and i.t. gallein was administered 30 minutes before morphine. Systemic NLX and NLXM were administered i.p. 15 minutes before morphine. I.c.v. NLXM was also administered 15 minutes before morphine.

### 2.4 Intracerebroventricular pretreatment injections

I.c.v. injections were performed 30 minutes after measurement of baseline withdrawal or response latencies and 30 minutes before morphine treatment. Hamilton syringes (10 *μ*L) with 26-G (Hamilton catalog #7804-03; point #4, 12° bevel) needles were used with a custom stopper that allowed 4 mm of the needle to enter the skull. Injections were performed freehand, using the ears and eyes for orientation to target the lateral ventricles of the brain. The volume of injection was 3 *μ*L for all experiments, and the needle was held in place for 30 seconds after the total volume was injected to ensure perfusion. After 30 seconds, the needle was carefully removed and the mice placed into a separate recovery cage and observed for recovery from anesthesia (normal grooming and movement). Upon recovery, mice were placed back into their home cage.

To conduct the injection, mice were anesthetized using isoflurane until no longer responsive to tail pinch and breathing slowed to 1 inhalation per second. After completion of the experiment, mice were euthanized to confirm injection placement using methylene blue.

### 2.5 Intrathecal pretreatment injections

One hour prior to habituation measurement in WWTW, mice were briefly anesthetized in isoflurane and the thoracolumbar region shaved using handheld electric trimmers. Mice were then placed back into home cages for recovery. I.t. injections were performed 30 minutes after measurement of baseline tail withdrawal latency and 30 minutes before morphine treatment. BD 3/10 mL insulin syringes with 5/16” x 31G Ultra-Fine needles (catalog #328438; Medline) were used to perform injections. Prior to injection, the mice were again briefly anesthetized. Subsequently, mice were gripped by one hand at the pelvic girdle to aid visual confirmation of spinous processes and a sterile needle inserted between the L4 and L5 vertebrae.^33^^,^^34^ Upon completion of injection, the needle was held in place for 30 seconds to prevent backflow. After 30 seconds, the needle was carefully removed and the mice placed into a separate recovery cage and observed for recovery from anesthesia (normal grooming and movement). Once recovered, mice were placed back into their home cage.

To confirm an adequate hit rate of i.t. injections prior to beginning experiments, separate cohorts of mice were administered i.t. injections of 3 *μ*g morphine and observed for a Straub tail to confirm injection placement. Upon obtaining >90% accuracy, experiments were performed using i.t. gallein.

### 2.6 WWTW

Mice were briefly placed into a cylindrical plexiglass restrainer and tails immersed ∼3 cm into a 55 °C water bath (50 °C for experiments using nalbuphine). The latency to withdraw or rapidly flick the tail was recorded with a maximum of 15 seconds (20 seconds for experiments using a 50 °C water bath; nalbuphine experiments) to prevent tissue damage. Baseline tail withdrawal latencies between 2 and 5 seconds were consistent for each assay. Mice were briefly habituated to the plexiglass restrainer and tail dipped before injection with saline. After 30 minutes, baseline withdrawal latencies were recorded.

For i.c.v. and i.t. gallein pretreatment experiments, withdrawal latencies were recorded 30 minutes after i.c.v. injection (Pre, pretreatment withdrawal latency). Pretreatment withdrawal latencies were recorded 15 minutes after the i.c.v. injection for i.c.v. NLXM pretreatment experiments. Mice were administered the analgesic drug immediately following the pretreatment measurement for gallein studies or immediately following the baseline measurement for dose-response studies involving a single analgesic drug alone. For time-course studies, withdrawal latencies were recorded every 15–30 minutes subsequent to MOR agonist, CP55940, or epibatidine treatment. For clonidine, latencies were recorded every 15 minutes for the first hour and every 30 minutes for the second hour. In dose-response curve studies, the interinjection interval was 30 minutes for buprenorphine, nalbuphine, CP55940, and epibatidine. A 15-minute interinjection interval was used for fentanyl and clonidine dose-response curves. Doses of MOR agonists, CP55940, clonidine, and epibatidine used for time-course studies were determined by selecting subeffective doses from dose-response curves for each agonist alone. For morphine, a dose of 3.2 mg/kg was selected based on previous work illustrating enhancement of antinociceptive effects by gallein at this dose.^14^

### 2.7 Hot plate

To measure response latencies in the hot plate assay, mice were placed onto a Hot Plate Analgesia Meter (Columbus Instruments) set to 52 °C and surrounded by 4 plexiglass walls. Response latency in the hot plate assay was measured as the time until licking of the hindpaws, flinching of the hindpaws, or escape/jumping behavior off the hot plate. The latency to responses was recorded with a maximum of 60 seconds to avoid tissue damage. Baseline latencies were consistent between 15 and 25 seconds. Mice were first habituated to the hot plate and injected with saline 30 minutes before the baseline measurement.

For gallein experiments, response latency was recorded 30 minutes after the i.c.v. injection (Pre, pretreatment response latency) and morphine was administered immediately thereafter. Subsequent time-course measurements were then taken every 30 minutes after morphine administration. Dose-response curve data of morphine on the hot plate assay used a 30-minute interinjection interval.

### 2.8 Locomotor activity

For locomotor activity studies, Opto-M3 Locomotor Activity Chambers (Columbus Instruments) were used. On day 1 (habituation), animals were habituated to locomotor activity chambers for 1 hour before receiving systemic gallein (i.p.) treatment and remained in the chamber for 3 hours. At 24 hours after gallein treatment (day 2), the mice were placed back into locomotor activity chambers and beam breaks (X-Y plane) recorded for 1 hour.

### 2.9 Statistics

For time-course studies and dose-response studies using gallein pretreatment, main effects and interactions were evaluated using repeated-measures analysis of variance in R with time or dose as a within-subject factor and sex, genotype, and treatments as between-subject factors as appropriate to the specific experiment. Post hoc analyses involved pairwise comparisons among groups at individual time points using the emmeans package with Tukey HSD adjustment. Additionally, to control for the overall family-wise error rate across total comparisons, global Bonferroni correction was applied. Study of sex-by-treatment interactions was not originally included in the original experimental design but emerged post hoc during data analysis.

Dose-response data using a single MOR agonist and no pretreatment condition were analyzed using a repeated-measures ANOVA with dose (baseline, dose 1, dose 2, dose 3, dose 4, and dose 5) as the within-subject factor and sex as the between-subject factor in R. Because no significant main effect of sex or sex-by-dose interaction was found, post hoc comparisons were subsequently performed using a Dunnett (treatment-vs-control) contrast computed from estimated marginal means obtained through the emmeans package in R. Baseline measurement served as the reference.

## 3 Results

### 3.1 Potentiation of morphine-induced antinociception by gallein requires activation of MORs

**Fig. 1:**
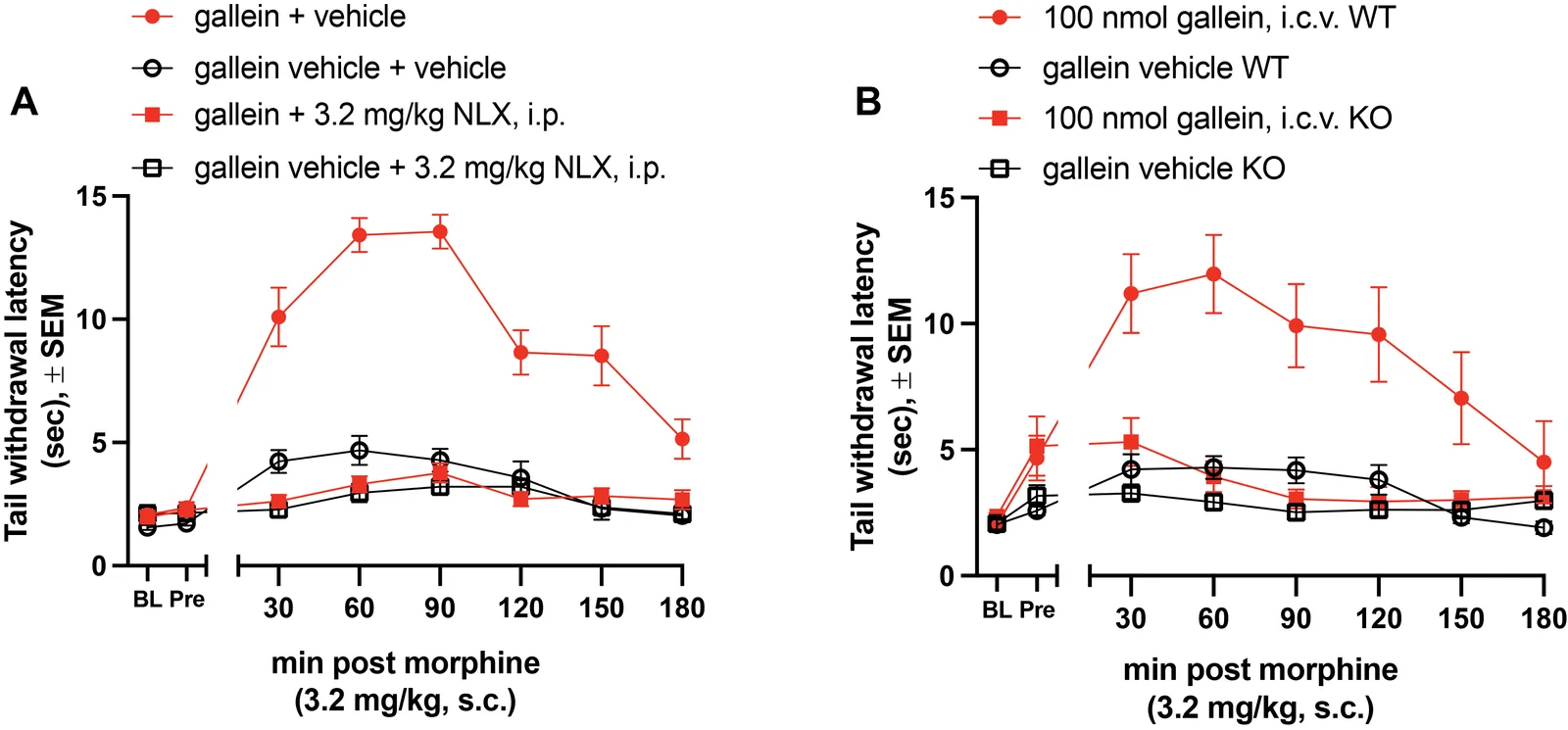
Enhancement of morphine-induced antinociception by gallein is dependent on morphine action at MOR. (A) There is a significant effect of 100 mg/kg gallein to enhance 3.2 mg/kg morphine-induced antinociception and this effect is blocked by 3.2 mg/kg naloxone (NLX) pretreatment (4-way repeated-measures analysis of variance; main effect of gallein pretreatment F [1, 41] = 52.33, *P* < .0001; main effect of NLX pretreatment F [1, 41] = 62.85, *P* < .0001; gallein pretreatment by NLX pretreatment interaction F [1, 41] = 42.60, *P* < .0001) (N = 16, 8 male/8 female, gallein + NLX vehicle; N = 12, 6 male/6 female, gallein + NLX; N = 11, 6 male/5 female, gallein vehicle + NLX; N = 10, 5 male/5 female, gallein vehicle + NLX vehicle) (post-test comparisons; Supplemental Table 1). (B) There is no significant effect of 100 nmol i.c.v. gallein to enhance 3.2 mg/kg morphine-induced antinociception in MOR knockout (KO) mice (3-way repeated-measures analysis of variance; main effect of gallein pretreatment F [1, 28] = 18.1428, *P* = .0002088; main effect of genotype F [1, 28] = 13.7496, *P* = .000914; gallein pretreatment by genotype interaction F [1, 28] = 9.3205, *P* = .004926) (N = 8, 4 male/4 female, vehicle wild type [WT]; N = 9, 5 male/4 female, gallein WT; N = 9, 5 male/4 female, vehicle KO; N = 10, 5 male/5 female, gallein KO). KO mice treated with gallein did not significantly differ from KO or WT mice treated with vehicle at any time point (post-test comparisons; Supplemental Table 2). BL, baseline; i.c.v., intracerebroventricular; i.p., intraperitoneal; MOR, *μ*-opioid receptor; Pre, pretreatment; s.c., subcutaneous.

Prior data support a model in which enhancement of morphine-induced antinociception by gallein depends on MORs, but this has not been formally demonstrated. To test this more directly, mice were systemically pretreated with gallein (100 mg/kg, i.p.) or an equivalent volume of a gallein vehicle 24 hours before morphine. Previously published data demonstrated that gallein delivered i.p. is effective up to 48 hours after administration,^32^ and Supplemental Figure 1 shows there are no effects on generalized locomotor activity by gallein after gallein administration in this paradigm. NLX (3.2 mg/kg, i.p.) or saline (NLX vehicle) was then administered 15 minutes prior to administration of a subanalgesic dose of morphine (3.2 mg/kg, subcutaneous [s.c.])^14^^,^^32^ (Fig. 1A). Tail withdrawal latencies were measured in the 55-°C WWTW assay. This dose of morphine alone had very little effect on tail withdrawal latencies compared with baseline. In accordance with prior findings,^14^^,^^30^^,^^32^ mice pretreated with gallein and saline (NLX vehicle) displayed significantly elevated morphine-dependent tail withdrawal latencies compared with morphine alone (gallein vehicle + NLX vehicle)—indicating potentiation of morphine-induced antinociception. However, when mice pretreated with gallein was followed by treatment with NLX, gallein enhancement of morphine-induced antinociception was blocked, indicating a requirement for morphine’s action at opioid receptors for gallein efficacy.

Considering that neither morphine nor NLX is highly selective for MOR relative to other opioid receptors, we investigated the effects of gallein on morphine-induced antinociception in MOR knockout (KO) mice and WT littermates (Fig. 1B). MOR KO mice and WT littermates received a 30-minute i.c.v. pretreatment with gallein (100 nmol) or vehicle prior to administration of morphine (3.2 mg/kg, s.c.) and assessment in the WWTW assay. This dose of i.c.v. gallein has previously been demonstrated to enhance morphine-induced antinociception,^14^ and also as shown previously, gallein alone does not alter tail withdrawal latencies over time in the absence of morphine (Supplemental Figure 2). Gallein-administered MOR KO mice did not exhibit elevated tail withdrawal latencies following morphine, whereas WT littermates did. Together, these results confirm a requirement for morphine action at MOR for gallein to enhance antinociceptive effects of morphine.

### 3.2 Gallein potentiates the antinociceptive effects of other MOR agonists

To evaluate the effect of gallein on MOR agonist--mediated antinociception with agonists other than morphine, we first determined appropriate doses of MOR agonists for each study. Dose-response curves were generated in separate cohorts of mice treated in the absence of gallein administration (Supplemental Figure 3). Both fentanyl and buprenorphine produced dose-dependent increases in tail withdrawal latencies in the WWTW assay, whereas nalbuphine only produced a significant increase in tail withdrawal latency at the highest dose tested.

**Fig. 2:**
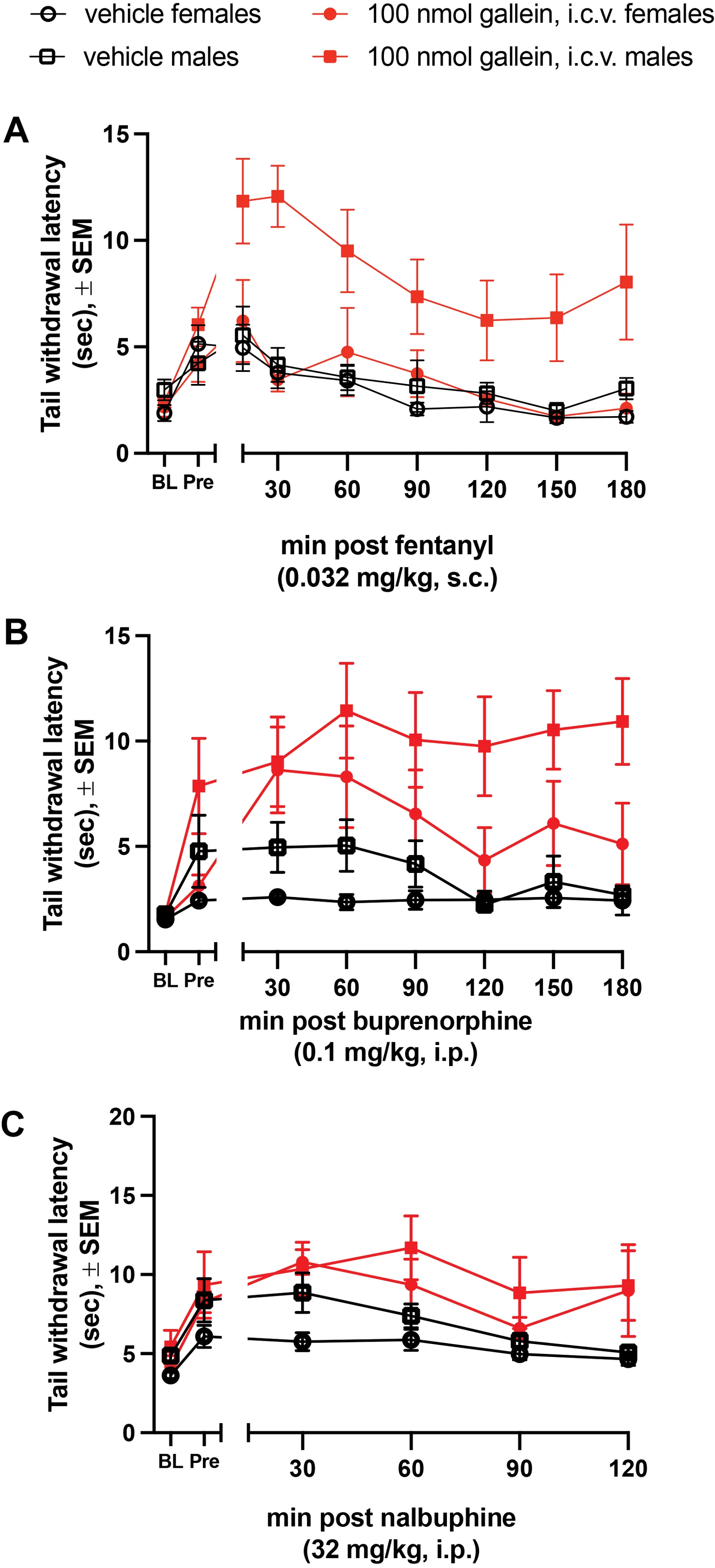
Gallein potentiates the antinociceptive effects of other *μ*-opioid receptor agonists in the warm water tail withdrawal assay. (A) Fentanyl-induced antinociception is enhanced by gallein in male mice, but not female mice (3-way repeated-measures analysis of variance [RM ANOVA]; main effect of sex F [1, 20] = 13.09, *P* = .0017; gallein pretreatment by sex interaction F [1, 20] = 8.117, *P* = .0099) (N = 6 vehicle female mice; N = 6 vehicle male mice; N = 6 gallein female mice; N = 6 gallein male mice). At the 30-minute timepoint, male mice treated with gallein exhibited significantly elevated tail withdrawal latencies (3-way RM ANOVA Tukey post-test with Bonferroni correction) compared with female mice treated with gallein (*P* = .0008) and compared with male mice treated with vehicle (*P* = .0025). (B) Pretreatment with 100 nmol i.c.v. gallein significantly enhances 0.1 mg/kg buprenorphine-induced antinociception (3-way RM ANOVA; main effect of gallein pretreatment F [1, 22] = 19.97, *P* = .0002; main effect of sex F [1, 22] = 6.269, *P* = .0202; no gallein pretreatment based on sex interaction F [1, 22] = 1.3467, *P* = .2583) (N = 14, 7 male/7 female, vehicle; N = 12, 6 male/6 female, gallein). Tail withdrawal latencies in gallein-pretreated mice were significantly higher than vehicle at every time point postbuprenorphine treatment (3-way RM ANOVA Tukey post-test with Bonferroni correction; 30 minutes *P* = .023, 60 minutes *P* = .01, 90 minutes *P* = .032, 120 minutes *P* = .014, 150 minutes *P* = .0095, and 180 minutes *P* = .005). (C) Pretreatment with 100 nmol i.c.v. gallein significantly enhances 32 mg/kg nalbuphine-induced antinociception (3-way RM ANOVA; main effect of gallein pretreatment F [1, 21] = 8.87, *P* = .0072) (N = 14, 7 male/7 female, vehicle; N = 11, 6 male/5 female, gallein). The gallein-pretreated group had significantly elevated tail withdrawal latencies (3-way RM ANOVA Tukey post-test with Bonferroni correction) compared with vehicle at 60 minutes (*P* = .0467). BL, baseline; i.c.v., intracerebroventricular; i.p., intraperitoneal; Pre, pretreatment; s.c., subcutaneous.

To test the effects of gallein on the responses to these MOR agonists, a single subanalgesic bolus dose of fentanyl, buprenorphine, or nalbuphine was used. Mice received a 30-minute i.c.v. pretreatment of gallein (100 nmol, i.c.v.) or vehicle, prior to administration of the agonist, followed by a time course in the WWTW assay. Gallein pretreatment significantly enhanced fentanyl-, buprenorphine-, and nalbuphine-induced antinociception as compared with vehicle pretreatment (Fig. 2A–C). Notably, there was a significant treatment by sex interaction in fentanyl experiments in which gallein significantly enhanced morphine-induced antinociception in male mice but not female mice (Fig. 2A). There was no significant treatment by sex interaction for buprenorphine (Fig. 2B) or nalbuphine (Fig. 2C), although qualitatively, there may be a sex interaction with gallein with these agonists as well.

### 3.3 Gallein enhances the effects of a cannabinoid receptor (CB1/2R) agonist and α2-adrenergic receptor agonist in a sex-dependent manner

**Fig. 3:**
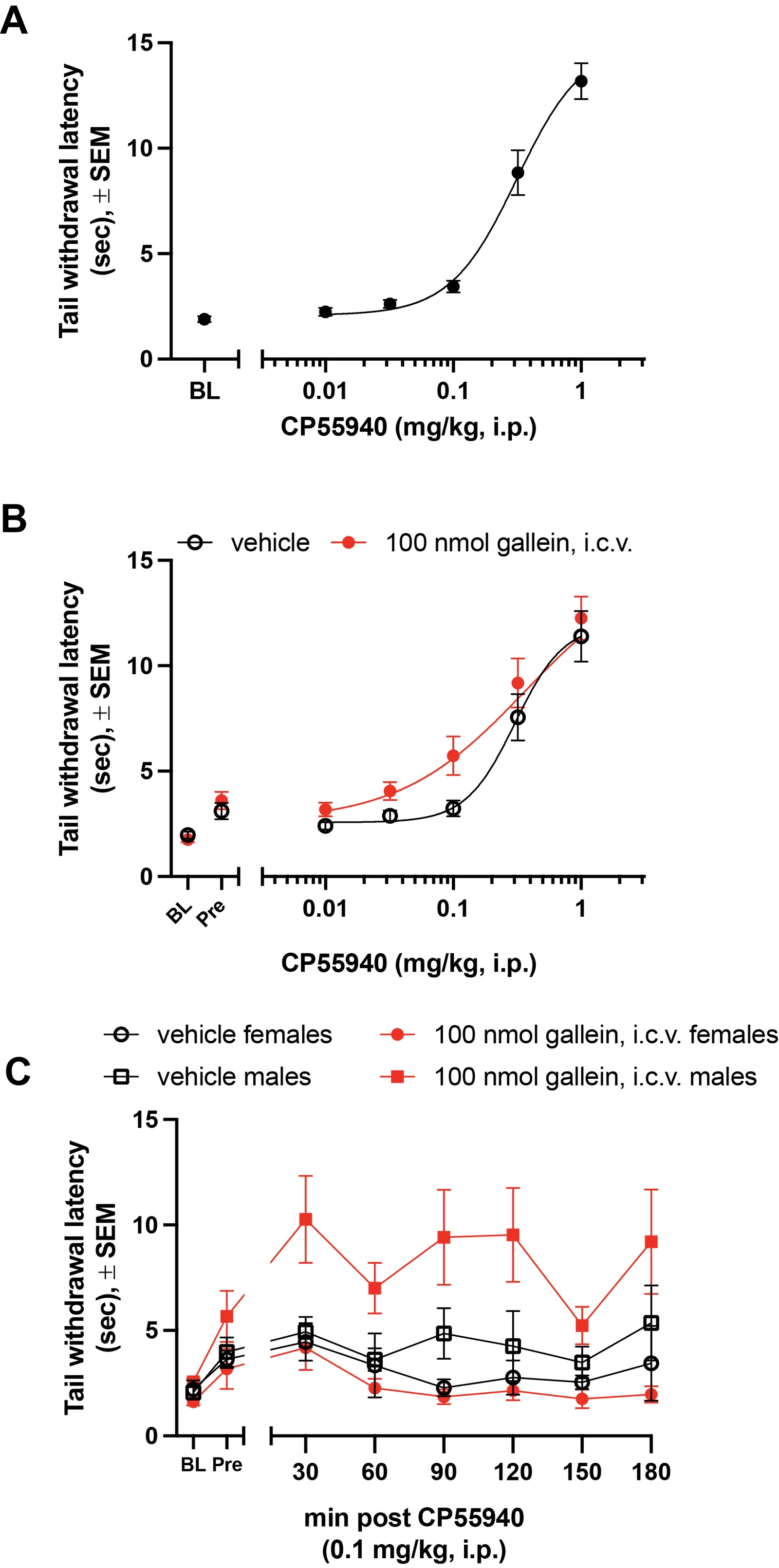
Gallein enhances antinociception mediated by a bolus dose of a cannabinoid receptor agonist, CP55940, in male mice. (A) CP55940 produces dose-dependent increases in tail withdrawal latencies (2-way repeated-measures analysis of variance [RM ANOVA]; main effect of dose F [5, 70] = 66.3341, *P* < .0001; no main effect of sex F [1, 14] = 0.7246, *P* = .409 or sex by dose interaction F [5, 70] = 0.4733, *P* = .795) (N = 16, 8 male/8 female). All doses 0.032 mg/kg or greater produced latencies significantly greater than baseline (Dunnett post-test). (B) Treatment with 100 nmol i.c.v. gallein does not significantly shift a dose-response curve of CP55940 in the warm water tail withdrawal assay (3-way RM ANOVA; main effect of dose F [6, 150] = 82.8825, *P* < .0001; no main effect of gallein pretreatment F [1, 25] = 1.6988, *P* = .2043; no main effect of sex F [1, 25] = 1.2045, *P* = .2829; no sex by gallein interaction F [1, 25] = 0.4941, *P* = .4886) (N = 14, 7 male/7 female, vehicle; N = 14, 7 male/7 female, gallein). (C) Treatment with 100 nmol i.c.v. gallein enhances 0.1 mg/kg CP55940-induced antinociception in male mice, but not female mice (3-way RM ANOVA; main effect of sex F [1, 19] = 12.308, *P* = .002351; gallein pretreatment by sex interaction F [1, 19] = 5.5386, *P* = .029522) (N = 5 vehicle female mice; N = 6 vehicle male mice; N = 6 gallein female mice; N = 6 gallein male mice). BL, baseline; i.c.v., intracerebroventricular; i.p., intraperitoneal; Pre, pretreatment.

Because G*βγ* signaling downstream to effectors such as PLC*β*3 arises primarily from Gi/o activation,^10^ gallein might be predicted to modulate G*βγ* signaling downstream of other antinociceptive Gi/o-coupled GPCRs to enhance their effects. We first evaluated cannabinoid receptors 1 and 2 (CB1/2Rs), which are both Gi/o coupled. We used CP55940, a CB1/2R agonist with greater potency and efficacy than tetrahydrocannabinol.^35^, ^36^, ^37^ A cumulative dose-response curve for CP55940 alone was used to determine a relatively equianalgesic dose of drug compared with previous opioid experiments (Fig. 3A). Animals were then pretreated with either 100 nmol i.c.v. gallein or vehicle before either cumulative dosing (Fig. 3B) or a single bolus dose of CP55940 (Fig. 3C). Although there was no main effect of gallein in either experiment, there was a significant effect of sex and gallein by sex interaction in the time-course studies driven by higher response latencies in male mice pretreated with gallein (Fig. 3C).

**Fig. 4:**
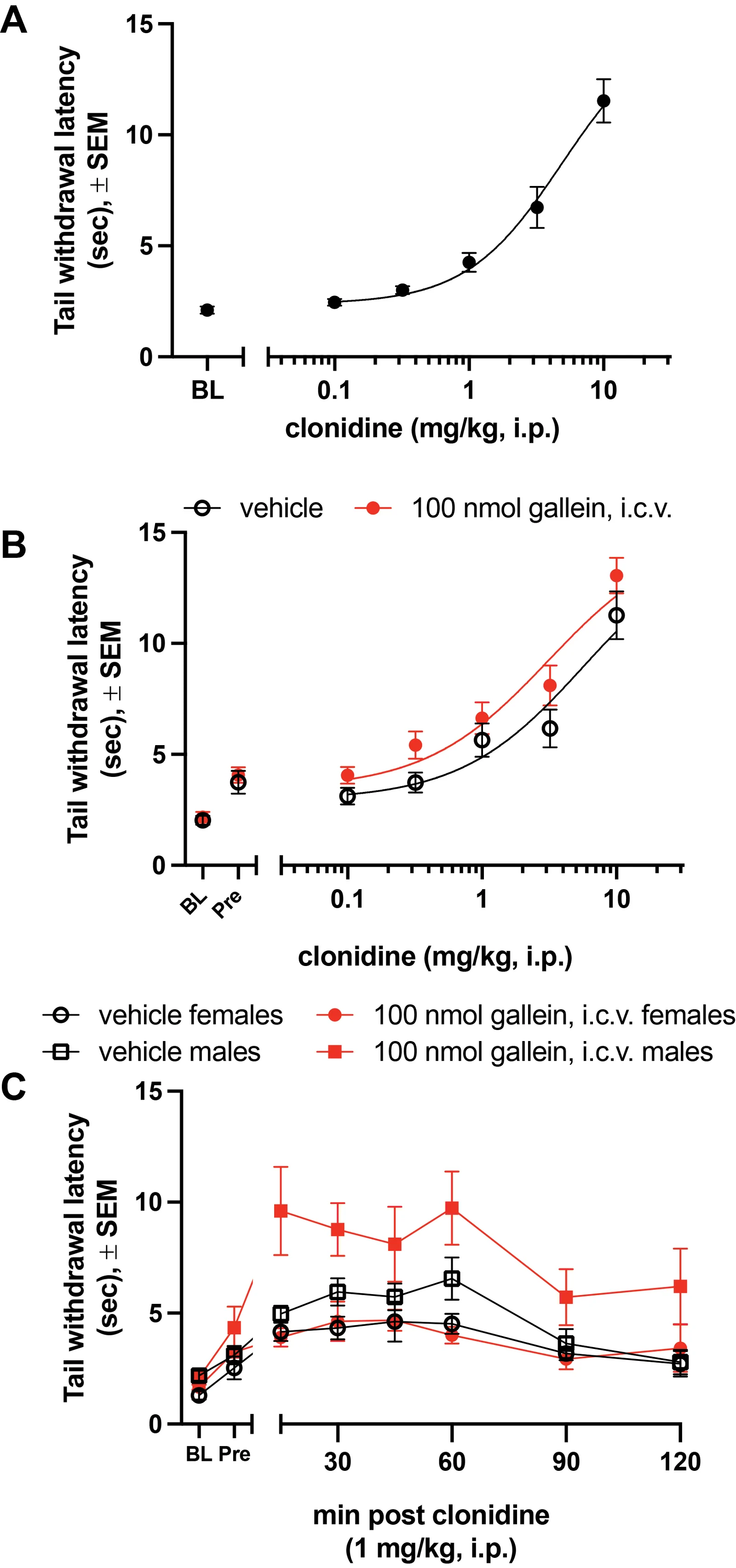
Gallein enhances antinociception mediated by a bolus dose of clonidine in male mice. **(**A) Clonidine produces a dose-dependent increase in tail-withdrawal latency (2-way repeated-measures analysis of variance [RM ANOVA]; main effect of dose F [5, 70] = 47.5651, *P* < .0001; no main effect of sex F [1, 14] = 1.0123, *P* = .3314; no sex by dose interaction F [5, 70] = 0.5781, *P* = .7165) with doses 0.32 mg/kg or greater, all significantly different from baseline (Dunnett post-test) (N = 16, 8 male/8 female). (B) Treatment with 100 nmol i.c.v. gallein did not significantly alter a dose-response curve of clonidine (3-way RM ANOVA; main effect of gallein F [1, 24] = 3.6064, *P* = .06964; main effect of dose F [6, 144] = 71.6202, *P* < .0001) (N = 14, 7 male/7 female, vehicle; N = 14, 7 male/7 female, gallein). (C) Treatment with 100 nmol i.c.v. gallein enhanced the antinociceptive effects of a bolus dose of 1 mg/kg clonidine (3-way RM ANOVA; main effect of gallein F [1, 21] = 7.8237, *P* = .0108) (N = 13, 7 male/6 female, vehicle; N = 13, 7 male/6 female, gallein). Enhancements in clonidine-induced antinociception by gallein are driven by elevated tail withdrawal latencies in male mice (3-way RM ANOVA; main effect of sex F [1, 21] = 17.7986, *P* = .000385; gallein pretreatment by sex interaction F [1, 21] = 6.4958, *P* = .018697) (N = 6 vehicle female mice, N = 7 vehicle male mice, N = 6 gallein female mice, N = 7 gallein male mice). BL, baseline; i.c.v., intracerebroventricular; i.p., intraperitoneal; Pre, pretreatment.

A similar set of experiments was conducted using the α2-adrenergic receptor (also Gi/o-coupled) agonist, clonidine. Although there was no effect of gallein treatment in dose-response studies (Fig. 4B), a main effect was observed in the time-course paradigm with a significant gallein by sex interaction (Fig. 4C). Although these effects do not appear as robust as those observed with MOR agonists, these data indicate that gallein potentiates antinociceptive signaling downstream of other Gi-coupled GPCRs.

### 3.4 Gallein does not enhance the antinociceptive effects of analgesics that do not rely on G protein signaling

**Fig. 5:**
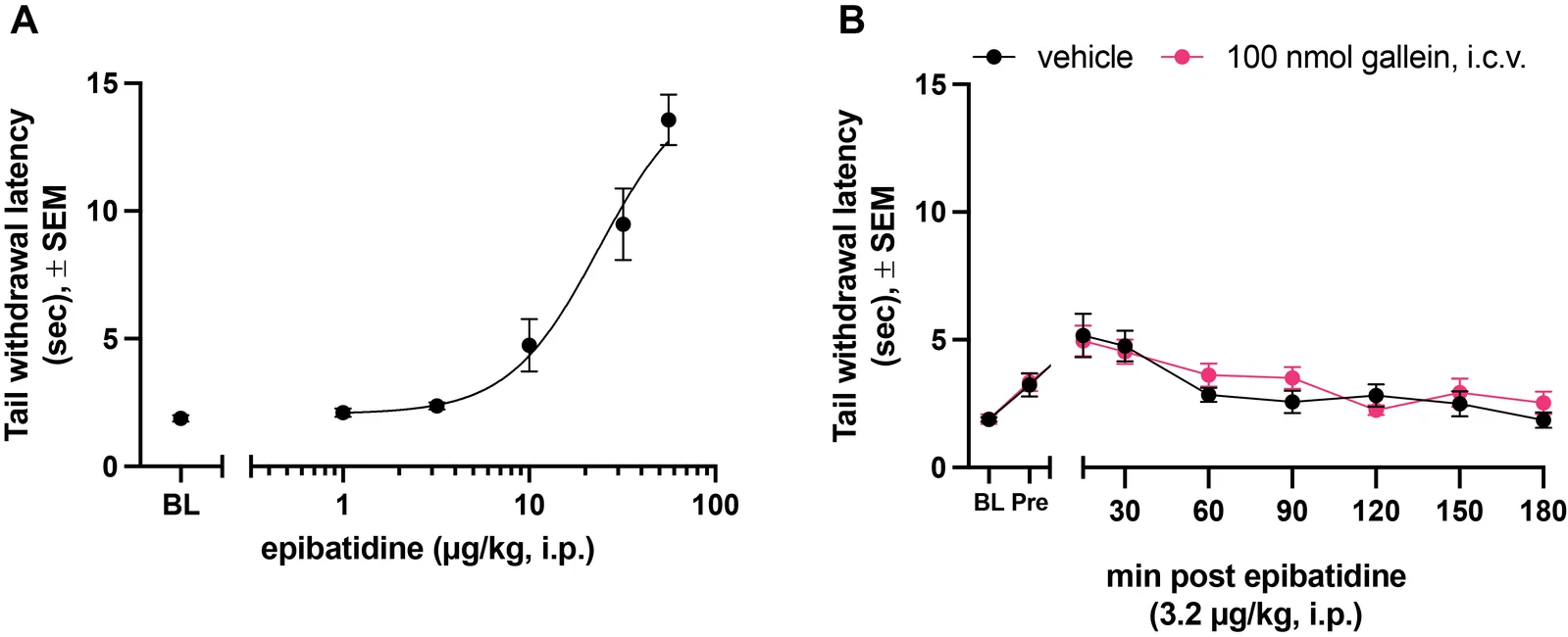
Gallein does not enhance epibatidine-induced antinociception. (A) Epibatidine produces dose-dependent increases in tail withdrawal latency (2-way repeated-measures analysis of variance; main effect of dose F [5, 60] = 40.6934, *P* < .0001; no main effect of sex F [1, 12] = 0.2091, *P* = .6556) (N = 14). Doses of 3.2 *μ*g/kg, 32 *μ*g/kg, and 56 *μ*g/kg were significantly different from baseline (Dunnett post-test). (B) Treatment with 100 nmol i.c.v. gallein did not enhance 3.2 *μ*g/kg epibatidine-induced antinociception (3-way repeated-measures analysis of variance; no main effect of gallein F [1, 20] = 0.4234, *P* = .5227) (N = 12, 7 male/7 female, vehicle; N = 12, 7 male/7 female, gallein). BL, baseline; i.c.v., intracerebroventricular; i.p., intraperitoneal; Pre, pretreatment.

Considering that gallein enhanced the antinociceptive effects of several distinct analgesics, it is important to further demonstrate that this is not a nonspecific effect on behavior. Prior research has shown that the nicotinic acetylcholine receptor agonist epibatidine potently elicits antinociception in rodents,^38^ and this effect was replicated in the WWTW assay in this study (Fig. 5A). Similar to previous experiments, mice were pretreated i.c.v. with 100 nmol gallein 30 minutes prior to a time course after epibatidine administration (Fig. 5B). There was no effect of gallein pretreatment on tail withdrawal latencies, demonstrating that gallein does not generally enhance antinociceptive responses.

### 3.5 Potentiation of morphine-induced antinociception by gallein requires activation of central but not peripheral MORs

**Fig. 6:**
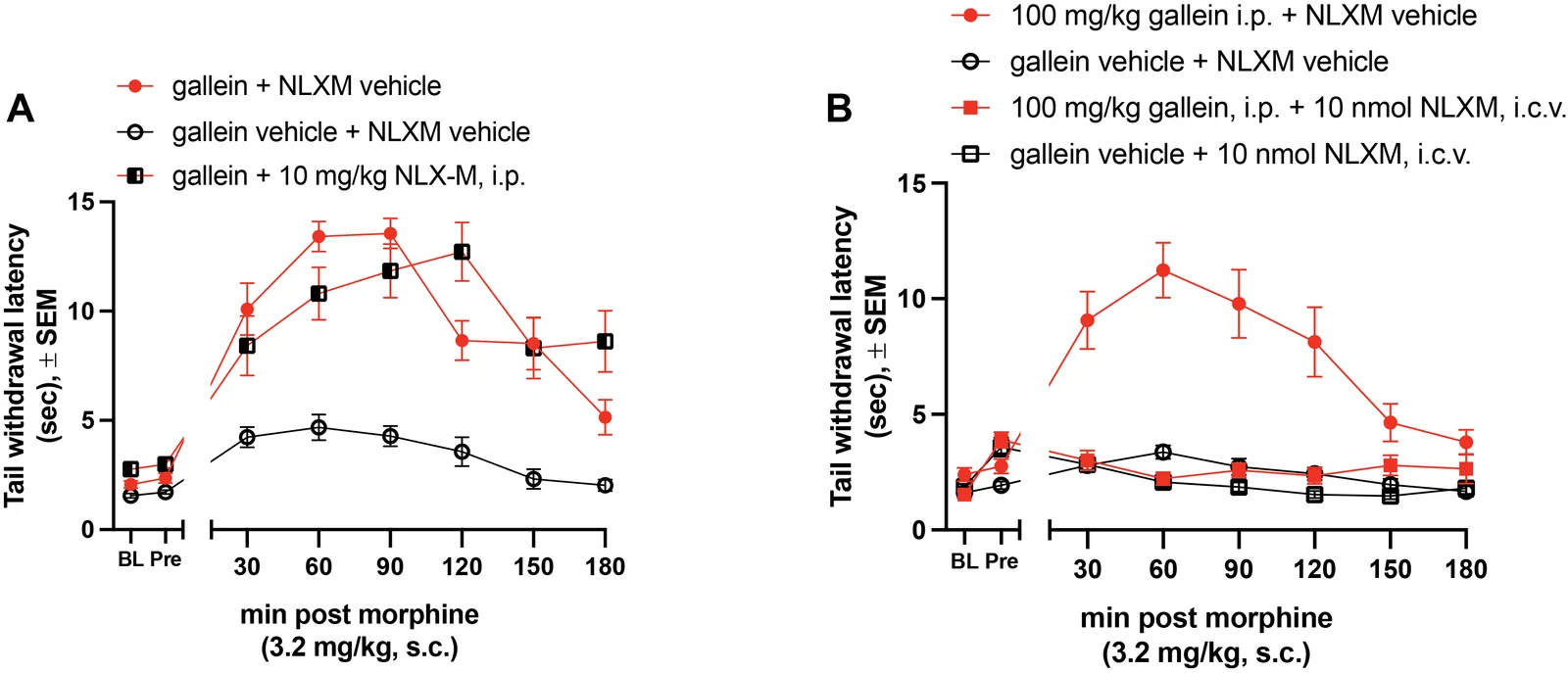
Enhancement of morphine-induced antinociception by gallein is dependent on morphine action at central *μ*-opioid receptors. (A) Enhancement of 3.2 mg/kg morphine-induced antinociception by 100 mg/kg i.p. gallein is not blocked by 10 mg/kg systemic naloxone methiodide (NLXM) (3-way repeated-measures analysis of variance; main effect of pretreatment condition F [2, 32] = 28.13, *P* < .0001). Post-test shows that mice treated with gallein and NLXM had elevated tail withdrawal latencies compared with mice treated with gallein vehicle although not being significantly different from mice treated with gallein and NLXM vehicle (N = 16, 8 male/8 female, gallein + NLXM vehicle; N = 12, 6 male/6 female, gallein + NLXM; N = 10, 5 male/5 female, gallein vehicle + NLX vehicle) (Supplemental Table 3). (B) When administered centrally, 10 nmol NLXM blocks enhancement of 3.2 mg/kg morphine-induced antinociception by 100 gallein mg/kg, i.p. (4-way repeated-measures analysis of variance; main effect of gallein pretreatment F [1, 40] = 43.52, *P* < .0001; i.c.v. NLXM pretreatment F [1, 40] = 32.58, *P* < .0001; gallein pretreatment by i.c.v. NLXM pretreatment interaction F [1, 40] = 26.87, *P* < .0001) (post-test comparisons; Supplemental Table 4) (N = 12, 6 male/6 female, gallein + vehicle; N = 12, 6 male/6 female, gallein + NLXM; N = 12, 6 male/6 female, gallein vehicle + vehicle; N = 12, 6 male/6 female, gallein vehicle + NLXM). BL, baseline; i.c.v., intracerebroventricular; i.p., intraperitoneal; Pre, pretreatment; s.c., subcutaneous.

To investigate whether it is morphine action at central or peripheral MORs involved in potentiation of antinociceptive effects by gallein, we utilized NLXM (a blood-brain barrier impermeable analog of NLX).^39^ Gallein or gallein vehicle was administered i.p. 24 hours prior to morphine. Fifteen minutes before morphine, mice received either NLXM (10 mg/kg, i.p.) to block peripheral MORs or saline (NLXM vehicle) (Fig. 6A). As is demonstrated in Fig. 1A, mice pretreated with gallein and saline exhibited significantly elevated tail withdrawal latencies as compared with mice pretreated with gallein vehicle and NLXM vehicle (this group of mice from Fig. 1A is again depicted here in Fig. 6A as open and closed circles). In contrast to NLX pretreatment, which blocked the effects of gallein, pretreatment with peripherally restricted NLXM did not (Fig. 6A; squares).

To support these data, we conducted a similar experiment in which mice were pretreated with systemic administration of gallein and central administration of NLXM (10 nmol, i.c.v.) (Fig. 6B). In contrast to the elevated tail withdrawal latencies observed with systemic gallein and NLXM, centrally administered NLXM blocked the effects of systemically administered gallein and morphine.

### 3.6 Central gallein potentiates morphine-induced antinociception in the hot plate assay

**Fig. 7:**
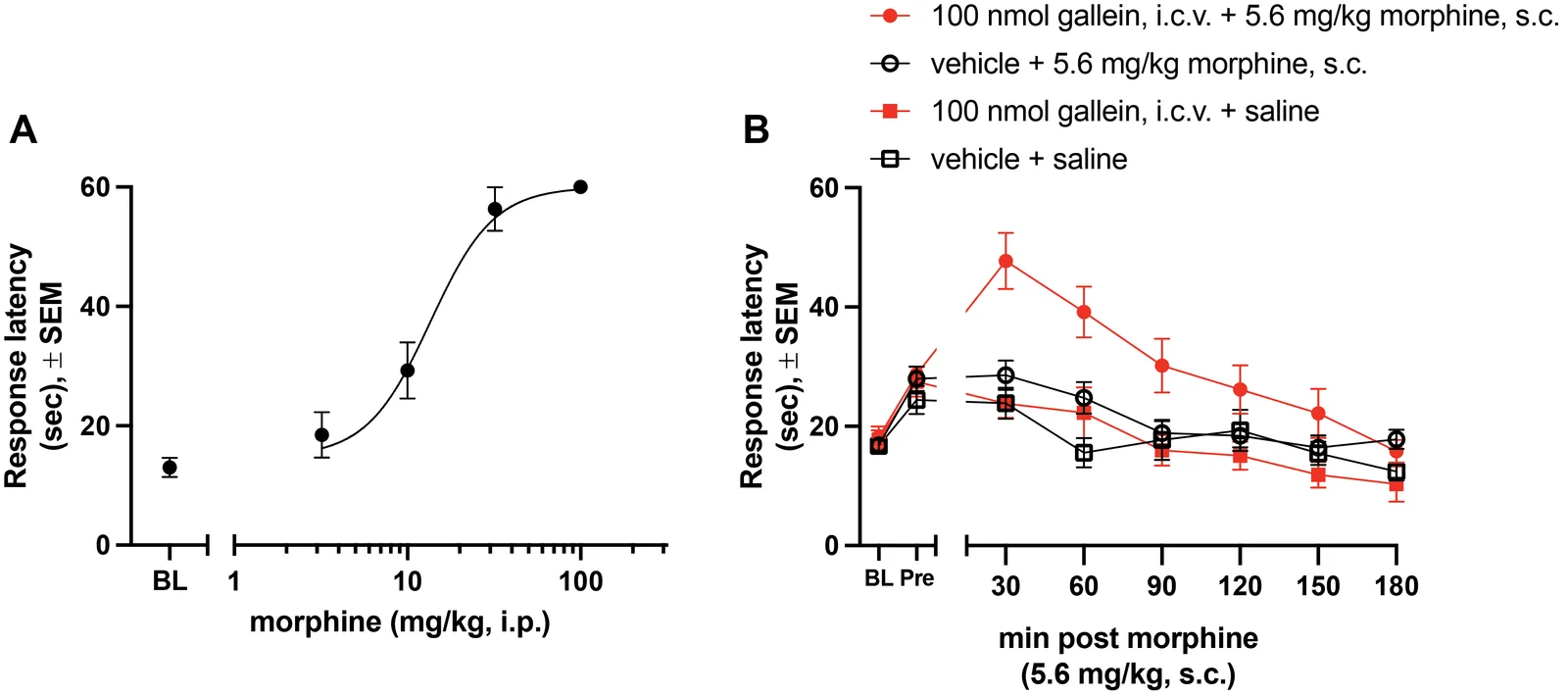
Gallein potentiates morphine-induced antinociception in the hot plate assay. (A) Morphine produces dose-dependent increases in tail withdrawal latency in the hot plate assay (2-way repeated-measures analysis of variance; main effect of dose F [4, 24] = 43.036, *P* < .0001). All doses greater than 3.2 mg/kg produce response latencies significantly above baseline (Dunnett post-test) (N = 8, 4 male/4 female). (B) Treatment with 100 nmol i.c.v. gallein significantly enhances morphine-induced antinociception in the hot plate assay (4-way repeated-measures analysis of variance; gallein pretreatment by morphine interaction F [1, 40] = 4.26, *P* = .0455; main effect of morphine F [1, 40] = 13.96, *P* = .0006) (post-test comparisons; Supplemental Table 5) (N = 12, 6 male/6 female, gallein vehicle + morphine; N = 12, 6 male/6 female, gallein + morphine; N = 12, 6 male/6 female, gallein vehicle + saline; N = 12, 6 male/6 female, gallein + saline). BL, baseline; i.c.v., intracerebroventricular; i.p., intraperitoneal; Pre, pretreatment; s.c., subcutaneous.

To further isolate whether the effects of gallein to enhance morphine-induced antinociception primarily occur at spinal or supraspinal MORs within the CNS, the effects of gallein were tested in the thermal antinociception hot plate assay. Responses observed in this assay are thought to be mediated supraspinally as compared with the spinal withdrawal reflex observed in WWTW. A dose-response curve for s.c. morphine was generated in this assay to determine the appropriate subanalgesic dose (Fig. 7A). Mice received i.c.v. gallein or vehicle pretreatment 30 minutes before morphine (5.6 mg/kg, s.c.) (Fig. 7B). Consistent with results observed in the WWTW assay, gallein pretreatment resulted in significantly elevated response latencies as compared with vehicle, whereas gallein alone had no effect. These data indicate that gallein enhances the antinociceptive effects of morphine in nociceptive assays other than the WWTW assay and that its effects may be due to action downstream of supraspinal MORs within the CNS.

### 3.7 Intrathecal gallein pretreatment did not enhance morphine-induced antinociception in the WWTW assay as compared to i.c.v. gallein pretreatment

**Fig. 8:**
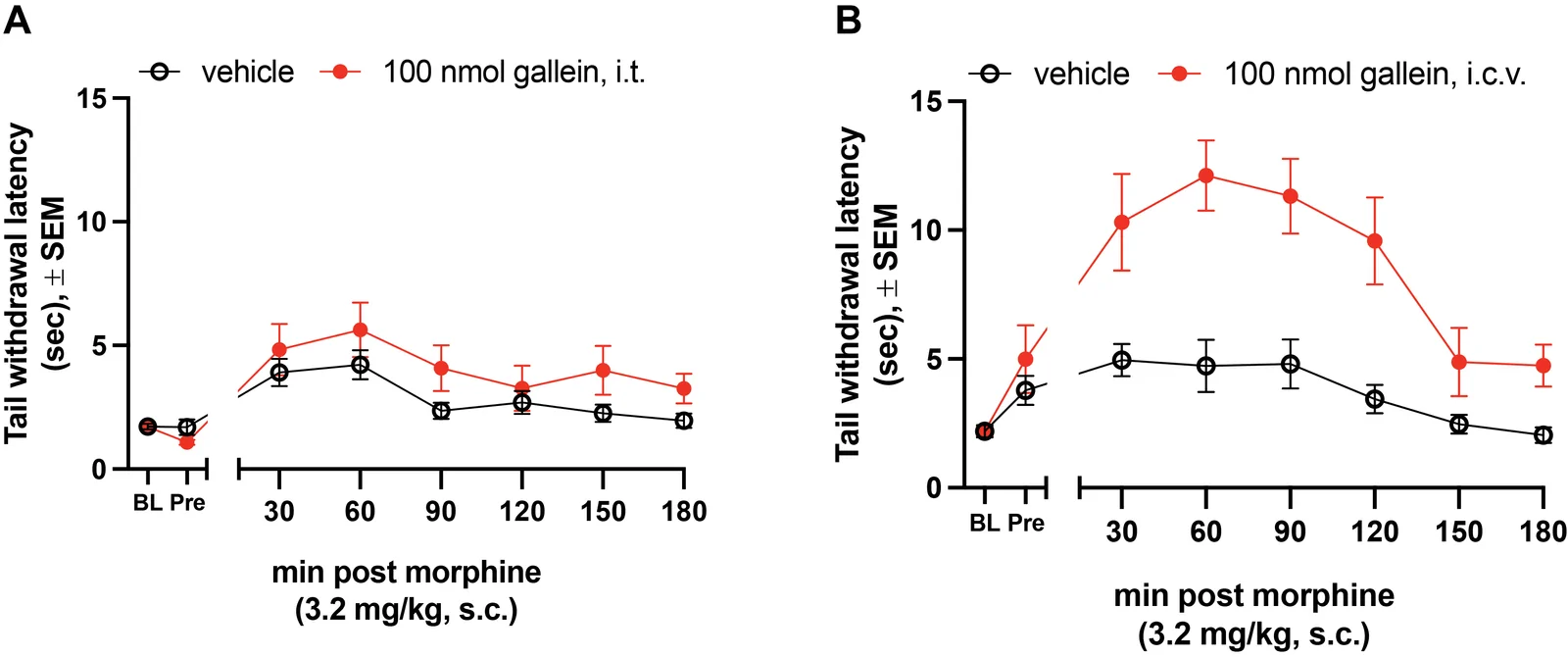
Intrathecal (i.t.) gallein pretreatment does not enhance morphine-induced antinociception, but i.c.v. gallein pretreatment does. (A) Antinociceptive effects of morphine were not enhanced by 100 nmol i.t. gallein (2-way repeated-measures analysis of variance; no main effect of gallein pretreatment F [1, 25] = 1.2892, *P* = .267) (N = 13, 5 male/8 female, vehicle; N = 16, 6 male/8 female, gallein). (B) Mice pretreated with 100 nmol i.c.v. gallein exhibit significantly elevated tail withdrawal latencies compared with vehicle-pretreated mice (2-way repeated-measures analysis of variance; main effect of gallein pretreatment F [1, 20] = 22.0584, *P* = .0001385; no main effect of sex F [1, 20] = 1.2268, *P* = .2812; no sex by gallein pretreatment interaction F [1, 20] = 0.0013, *P* = .9716) (post-test comparisons; Supplemental Table 6) (N = 8, 4 male/4 female, vehicle; N = 9, 4 male/5 female, gallein). BL, baseline; i.c.v., intracerebroventricular; Pre, pretreatment; s.c., subcutaneous.

To complement data observed in the hot plate assay, which suggest an effect of gallein at supraspinal MORs, studies were conducted in which mice were pretreated with gallein via the i.t. route. These injections are expected to remain relatively local without substantial gallein concentrations in the brain.^40^, ^41^, ^42^ Mice pretreated with i.t. gallein 30 minutes prior to morphine exhibited tail withdrawal latencies that were no different compared with vehicle-pretreated mice (Fig. 8A). In contrast, mice pretreated with i.c.v. gallein exhibited significantly elevated tail withdrawal latencies compared with vehicle-pretreated mice (Fig. 8B). These data corroborate hot plate findings that implicate enhancement of signaling downstream of supraspinal MORs for gallein to enhance morphine-induced antinociception.

### 3.8 Gallein does not potentiate morphine-induced antinociception in MOR 10S/T-A knock-in mice lacking MOR GRK and PKC phospho-sites

**Fig. 9:**
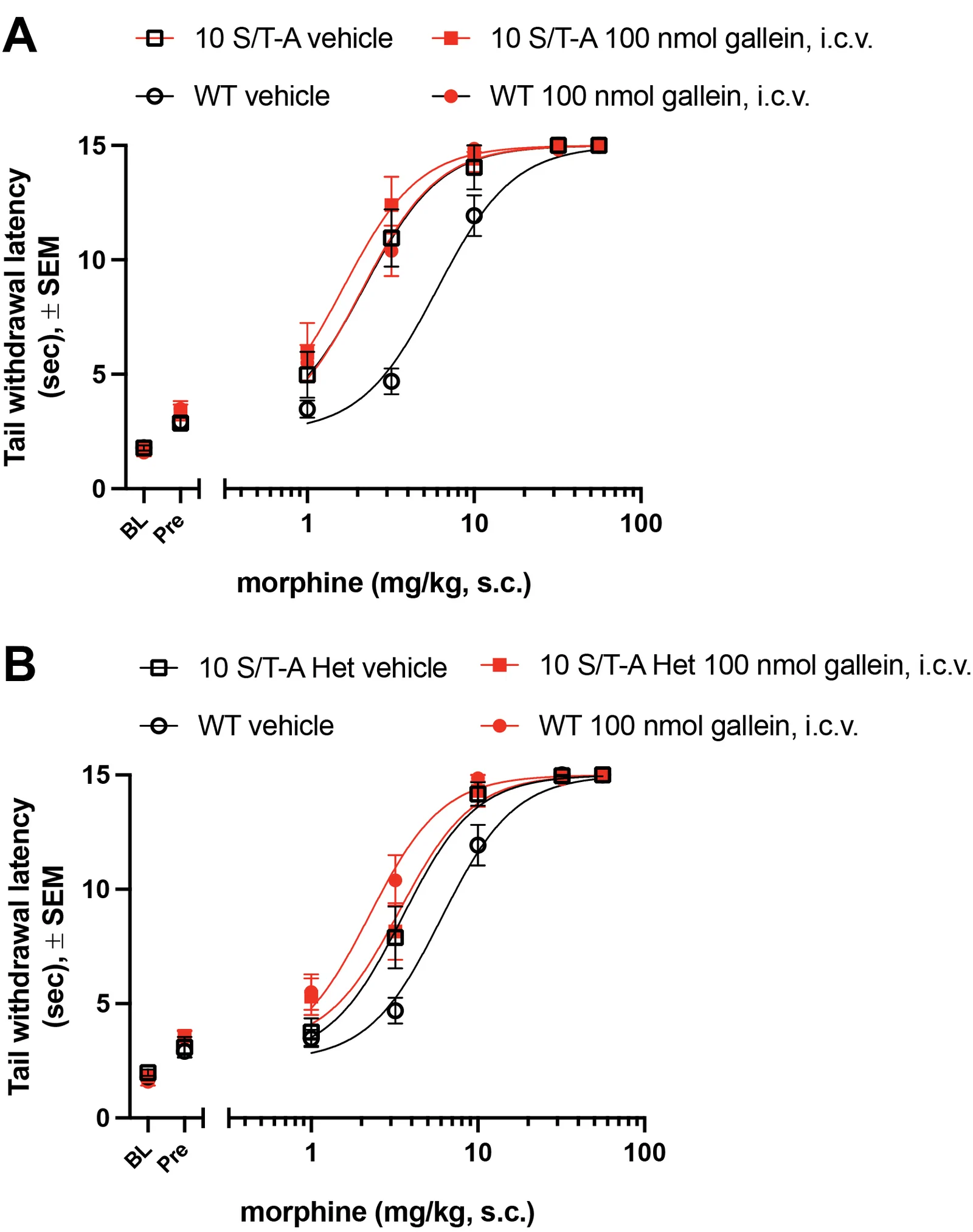
Gallein does not potentiate morphine-induced antinociception in mice expressing *μ*-opioid receptors lacking phosphorylation in the carboxyl terminus (10 S/T-A mice). (A) Treatment with 100 nmol i.c.v. gallein produced a significant leftward shift in the dose-response curve for morphine, and the effect of gallein on the dose-response relationship of morphine was significantly altered in homozygous 10 S/T-A knock-in mice (3-way repeated-measures analysis of variance; main effect of gallein F [1, 52] = 9.8159, *P* = .00284; main effect of genotype F [1, 52] = 7.1717, *P* = .00989; gallein by dose interaction F [6, 312] = 6.1542, *P* < .0001; gallein by dose by genotype interaction F [6, 312] = 2.5217, *P* = .0213) (N = 16, 8 male/8 female, wild-type [WT] vehicle; N = 16, 8 male/8 female, WT gallein; N = 12, 6 male/6 female, 10 S/T-A vehicle; N = 12, 6 male/6 female, 10 S/T-A gallein). At 3.2 mg/kg morphine, WT animals treated with gallein (*P* = .00485), 10 S/T-A mice treated with vehicle (*P* = .00392), and 10 S/T-A mice treated with gallein (*P* = .000147) all had significantly elevated tail withdrawal latencies compared with WT animals treated with vehicle but did not differ from each other. (B) Treatment with 100 nmol i.c.v. gallein did not alter a dose-response curve of morphine in heterozygous 10 S/T-A mice (2-way repeated-measures analysis of variance; no main effect of gallein pretreatment F [1, 30] = 0.517, *P* = .478; no gallein pretreatment by dose interaction F [6, 180] = 0.505, *P* = .804) (N = 16, 8 male/8 female, 10 S/T-A Het vehicle; N = 16, 8 male/ 8 female, 10 S/T-A Het gallein). BL, baseline; i.c.v., intracerebroventricular; Pre, pretreatment; s.c., subcutaneous.

A central mechanistic hypothesis is that gallein exerts its effects on MOR by preventing G*βγ*-mediated activation of effectors such as GRK and PLC*β*3 that inhibit MOR function. As a test of this in vivo, we utilized knock in mice in which 10 critical GRK and PKC phosphorylation sites in MOR were mutated from serine/threonine to alanine (MOR 10S/T-A mice).^43^ MORs in these animals cannot be phosphorylated at the C-terminal tail by GRK or PKC. If gallein acts by preventing activation of GRK- and PKC-dependent phosphorylation of MORs, gallein would be expected to have a minimal effect in these animals. Homozygous MOR 10S/T-A and WT littermates were pretreated with 100 nmol gallein, i.c.v. or vehicle, before a dose-response analysis of morphine in the WWTW assay (Fig. 9A and Table 1). As previously shown, WT mice pretreated with gallein exhibited a significant leftward shift in the dose-response curve for the antinociceptive effects of morphine compared with vehicle-pretreated controls. Also, as previously published, 10S/T-A mice also exhibited a significant leftward shift compared with WT in the absence of gallein treatment.^43^ No shift was observed in 10S/T-A littermates pretreated with gallein compared with vehicle. Surprisingly, gallein effects were also eliminated in heterozygous 10S/T-A mice (Fig. 9B and Table 1). Overall, these data support the proposed model by indicating that the action of gallein requires the ability to modulate the phosphorylation state of C terminal amino acids on MOR by PKC/GRK2.

**Table 1:** EC_50_ values for responses of WT, 10 S/T-A homozygous, and 10 S/T-A heterozygous mice to gallein treatment

Genotype/treatment	Log EC_50_ morphine (mg/Kg)	95% CI	N
WT Veh	0.78	0.70-0.85	16
WT Gallein	0.34	0.25-0.43	16
10 S/T-A Veh	0.33	0.23-0.42	12
10 S/T-A Gallein	0.21	0.12-0.31	12
10 S/T-A-het Veh	0.55	0.47-0.62	16
10 S/T-A-het Gallein	0.53	0.44-0.62	16

WT, wild type.

## 4 Discussion

There is an ever-increasing body of evidence supporting pharmacological modulation of G*βγ* subunit signaling with gallein downstream of Gi-coupled GPCRs as an efficacious therapeutic approach in preclinical models. Gallein efficacy has been demonstrated in multiple chronic disease models including mouse models of lupus, heart failure, cardiac fibrosis, and atherosclerosis^23^, ^24^, ^25^^,^^44^^,^^45^ without overt side effects. There is also strong evidence that pharmacological manipulation of G*βγ* signaling downstream of MOR activation with M119 or gallein enhances the antinociceptive effects of morphine.^14^^,^^19^^,^^22^^,^^30^^,^^32^ Importantly, these enhancements in morphine-induced antinociception occur in the absence of common opioid-related side effects such as respiratory depression and constipation.^30^ More recent investigation has demonstrated that gallein treatment inhibits the development of tolerance to the antinociceptive effects of morphine and enhances morphine-induced antinociception in a tolerant state.^32^ In this previous study, it was found that gallein efficacy persisted up to 48 hours after administration. We do not understand the mechanism for the long duration of action, which was confirmed in this work, and also noted in a previous study.^44^ We also recently demonstrated that gallein enhances the antinociceptive effects of endogenous opioids in a model of chronic pain.^31^

Despite the accumulating evidence, it remained possible that the in vivo effects of gallein were unrelated to its ability to modulate MOR activity. In this study, we formally show that the ability of gallein to enhance the antinociceptive effects of morphine depends on morphine action at MORs. Further, gallein enhances the antinociceptive effects of other opioid analgesics fentanyl, buprenorphine, and nalbuphine. The fact that the effects of gallein are generalizable across different MOR agonists indicates that the actions of gallein are not attributable to a specific idiosyncratic interaction of gallein and morphine (eg, altering metabolism).

We also showed that enhancements in morphine-induced antinociception by gallein specifically depended on morphine action at central, but not peripheral, MORs. This is evidenced by data showing NLXM only prevents the effects of gallein when administered centrally. Hot plate experiments (which measure supraspinally mediated behaviors) and i.t. administration of gallein support this conclusion. These results are consistent with the efficacy of gallein when administered i.c.v. and suggest that gallein directly influences agonist potency at presynaptic MORs in isolated periaqueductal gray slices.^14^

We predicted that gallein might enhance the activity of any presynaptic Gi/o-coupled GPCR. This prediction was based on the observation that the majority of G*β*γ-mediated signal transduction, including activation of PLC*β*3, is downstream of Gi-coupled GPCRs owing to sensitivity of these responses to blockade by pertussis toxin. Indeed, we did observe effects of gallein on antinociception driven by CB1/2 (CP55940) or α2ARs (clonidine), 2 Gi-coupled receptors, but not for stimulation by the nicotinic acetylcholine receptor agonist epibatidine. Gallein-dependent enhancement of the effects of these agonists was not as large as that seen for morphine (and only in male mice, see below). Because gallein does not act directly on GPCRs but rather acts on the signaling pathways downstream, its effects are dependent both on the cellular context and the specific mechanisms mediating feedback for that particular GPCR, ultimately leading to differences in gallein responses. Additionally, the circuit mechanisms utilized by CB1/2 or *α*2ARs to mediate analgesia are likely very different from those utilized by MORs that could also lead to differences in gallein effects. Overall, these results support the notion that gallein can influence the actions of other nonopioid Gi/o-coupled receptor systems.

Sex differences were not an explicit area of investigation in part because they had not been previously observed for gallein interactions with morphine and as shown in Fig. 1. There was, however, a significant difference in the ability of gallein to potentiate fentanyl-, CP55940-, and clonidine-induced antinociception between male and female mice. Considering there were no sex differences observed in antinociceptive effects for mice administered fentanyl, CP55940, or clonidine alone, it is possible that there are sex-specific responses to the effects of gallein. This could be due to sex differences in expression and/or activity of various G*βγ* effectors that are inhibited by gallein.^46^, ^47^, ^48^ Nevertheless, this cannot explain why gallein-dependent sex effects were seen for some agonists (eg, fentanyl) but not others (morphine). Further work must be done to reach concrete conclusions regarding potential mechanisms for sex differences in the effects of gallein.

Finally, experiments utilizing 10 S/T-A mice, which express MORs that cannot undergo C-terminal phosphorylation by GRK or PKC,^43^ strongly support the proposed mechanism of gallein action in an in vivo mouse model. Given that MORs cannot be directly regulated by GRKs or PKC in these mice, it would be expected, based on our model, that gallein would be ineffective in these mice. Homozygous 10 S/T-A, and surprisingly heterozygous, mice did not respond to gallein in the WWTW assay. These data provide mechanistic in vivo evidence that gallein potentiates morphine-induced antinociception by abrogating G*βγ*-mediated activation of GRK2 and PLC*β*3 and subsequent MOR phosphorylation.

Together, these data support the proposed model in which the “tool compound” gallein enhances opioid potency at neurons in the CNS by selectively inhibiting Gβγ interactions with enzymes involved in negative feedback regulation of MORs, whereas at the same time preserving G*βγ* interactions with ion channels and/or soluble N-ethylmaleimide-sensitive factor attachment receptors required for MOR antinociceptive actions. The preponderance of data suggests that this overall approach has therapeutic potential and incentivizes ongoing work to develop more “drug like” molecules that behave similarly to M119 and gallein.

## Conflict of interest

The authors declare no conflicts of interest.
