# Supplementary material for: Mechanisms for enhancement of μ-opioid receptor-mediated antinociception in the central nervous system through gallein-dependent modulation of downstream G protein βγ subunit signaling
Source: Mol Pharmacol. 2026 Apr 20;108(5-6):100123. doi: 10.1016/j.molpha.2026.100123 (PMC13494096; doi:10.1016/j.molpha.2026.100123)
Supplement: Supplementary file 1 [file mmc1.docx]

Mechanisms for Enhancement of μ-Opioid Receptor-Mediated Antinociception in the Central Nervous System Through Gallein-Dependent Modulation of Downstream G Protein βγ Subunit Signaling

Jacob A. Ormes, Alan V. Smrcka^#^ and Emily M. Jutkiewicz

Department of Pharmacology, University of Michigan, Ann Arbor, MI 48109

**Molecular Pharmacology**

Supplemental Information

Supplemental Figures and Legends:

**Supplemental Figure 1. Systemic gallein does not alter locomotor activity 24 hrs post administration.** Total beam breaks over the course of 1 hr are not different between gallein and vehicle pretreated groups 24 hrs post administration (Unpaired t-test T (30) = 1.098, P=0.2807) (N=16 vehicle, N=16 gallein).

**Supplemental Figure 2. Gallein has no effect on antinociception in the absence of morphine.** Tail withdrawal latencies do not differ in mice pretreated with gallein or vehicle prior to a time course of saline in the WWTW assay (Two-way RM ANOVA, no main effect of gallein pretreatment (F(1, 19) = 0.1401, P=0.712) (N=12 vehicle, N=11 gallein).

**Supplemental Figure 3. Dose response curves for fentanyl, buprenorphine, and nalbuphine. A)** Fentanyl produces dose-dependent increases in tail withdrawal latency (Two-way RM ANOVA, main effect of dose F(5, 30) = 108.1, P<0.0001; no main effect of sex F(1, 6) = 2.9861, P=0.1347; no sex by dose interaction F(5, 30) = 2.2826, P=0.0715) (N=8, 4 male/4 female). Doses 0.01 mg/kg (P=0.043), 0.032 mg/kg (P=0.0347), 0.32 mg/kg (P<0.0001), and 1 mg/kg (P<0.0001) are significantly different from baseline (Dunnett’s post-test). **B)** Buprenorphine produces dose-dependent increases in tail withdrawal latency (2-way RM ANOVA, main effect of dose F(5, 45) = 90.1, P<0.0001; no main effect of sex F(1, 9) = 0.6431, P=0.4432; no sex by dose interaction F(5, 45) = 0.1957, P=0.9626) (N=11, 6 male/5 female). Doses 0.32 mg/kg (P=0.0054), 1 mg/kg (P=0.0004), and 3.2 mg/kg (P<0.0001) are significantly different from baseline (Dunnett’s post-test). **C)** Nalbuphine produces antinociception in the WWTW assay at 50°C (2-way RM ANOVA, main effect of dose F (5, 30) = 4.38, P=0.0407; no main effect of sex F(1, 6) = 0.0485, P=0.8330; no sex by time interaction F(5, 30) = 1.4674, P=0.2297) (N=8, 4 male/4 female). Only the largest 320 mg/kg (P=0.0018) dose is significantly different from baseline (Dunnett’s post-test).

| **Comparison** | **Time (min)** | **p-value** |
| --- | --- | --- |
| gallein + NLX vehicle vs gallein + NLX | 30 | <0.0001 |
| gallein + NLX vehicle vs gallein vehicle + NLX vehicle | 30 | 0.0036 |
| gallein + NLX vehicle vs gallein vehicle + NLX | 30 | <0.0001 |
| gallein + NLX vehicle vs gallein + NLX | 60 | <0.0001 |
| gallein + NLX vehicle vs gallein vehicle + NLX vehicle | 60 | <0.0001 |
| gallein + NLX vehicle vs gallein vehicle + NLX | 60 | <0.0001 |
| gallein + NLX vehicle vs gallein + NLX | 90 | <0.0001 |
| gallein + NLX vehicle vs gallein vehicle + NLX vehicle | 90 | <0.0001 |
| gallein + NLX vehicle vs gallein vehicle + NLX | 90 | <0.0001 |
| gallein + NLX vehicle vs gallein + NLX | 120 | <0.0001 |
| gallein + NLX vehicle vs gallein vehicle + NLX vehicle | 120 | 0.000637 |
| gallein + NLX vehicle vs gallein vehicle + NLX | 120 | 0.000124 |
| gallein + NLX vehicle vs gallein + NLX | 150 | 0.00417 |
| gallein + NLX vehicle vs gallein vehicle + NLX vehicle | 150 | 0.00404 |
| gallein + NLX vehicle vs gallein vehicle + NLX | 150 | 0.00171 |

**Post-test Comparison Tables**

Post-test comparisons are adjusted by Tukey HSD within each time point and Bonferroni adjusted by the number of timepoint comparisons to control family-wise error.

**Supplemental Table 1. Figure 1A Post-test Comparisons**

**Supplemental Table 2. Figure 1B Post-test Comparisons**

| **Comparison** | **Time (min)** | **p-value** |
| --- | --- | --- |
| gallein WT vs vehicle WT | 30 | 0.00250 |
| gallein WT vs vehicle KO | 30 | 0.000257 |
| gallein WT vs gallein KO | 30 | 0.00538 |
| gallein WT vs vehicle WT | 60 | <0.0001 |
| gallein WT vs vehicle KO | 60 | <0.0001 |
| gallein WT vs gallein KO | 60 | <0.0001 |
| gallein WT vs vehicle WT | 90 | 0.014 |
| gallein WT vs vehicle KO | 90 | 0.000311 |
| gallein WT vs gallein KO | 90 | 0.000555 |
| gallein WT vs vehicle KO | 120 | 0.00543 |
| gallein WT vs gallein KO | 120 | 0.00744 |

**Supplemental Table 3. Figure 6A Post-test Comparisons**

| **Comparison** | **Time (min)** | **p-value** |
| --- | --- | --- |
| gallein + NLXM vs gallein vehicle + NLXM vehicle | 0 | 0.009 |
| gallein + NLXM vs gallein vehicle + NLXM vehicle | 60 | 0.016 |
| gallein + NLXM vehicle vs gallein vehicle + NLXM vehicle | 60 | <0.0001 |
| gallein + NLXM vs gallein vehicle + NLXM vehicle | 90 | 0.003 |
| gallein + NLXM vehicle vs gallein vehicle + NLXM vehicle | 90 | <0.0001 |
| gallein + NLXM vs gallein vehicle + NLXM vehicle | 120 | <0.0001 |
| gallein + NLXM vs gallein vehicle + NLXM vehicle | 180 | 0.011 |

**Supplemental Table 4. Figure 6B Post-test Comparisons**

| **Comparison** | **Time (min)** | **p-value** |
| --- | --- | --- |
| gallein + NLXM vehicle vs gallein vehicle + NLXM vehicle | 30 | <0.0001 |
| gallein + NLXM vehicle vs gallein vehicle + NLXM | 30 | <0.0001 |
| gallein + NLXM vehicle vs gallein + NLXM | 30 | 0.00012 |
| gallein + NLXM vehicle vs gallein vehicle + NLXM vehicle | 60 | <0.0001 |
| gallein + NLXM vehicle vs gallein vehicle + NLXM | 60 | <0.0001 |
| gallein + NLXM vehicle vs gallein + NLXM | 60 | <0.0001 |
| gallein + NLXM vehicle vs gallein vehicle + NLXM vehicle | 90 | <0.0001 |
| gallein + NLXM vehicle vs gallein vehicle + NLXM | 90 | <0.0001 |
| gallein + NLXM vehicle vs gallein + NLXM | 90 | <0.0001 |
| gallein + NLXM vehicle vs gallein vehicle + NLXM vehicle | 120 | 0.0032 |
| gallein + NLXM vehicle vs gallein vehicle + NLXM | 120 | 0.00026 |
| gallein + NLXM vehicle vs gallein + NLXM | 120 | 0.0025 |
| gallein + NLXM vehicle vs gallein vehicle + NLXM | 150 | 0.0102 |

**Supplemental Table 5. Figure 7B Post-test Comparisons**

| **Comparison** | **Time (min)** | **p-value** |
| --- | --- | --- |
| gallein + morphine vs gallein vehicle + morphine | 30 | 0.046 |
| gallein + morphine vs gallein + saline | 30 | 0.002 |
| gallein + morphine vs gallein vehicle + saline | 30 | 0.002 |
| gallein + morphine vs gallein vehicle + saline | 60 | 0.009 |

**Supplemental Table 6. Figure 8B Post-test Comparisons**

| **Comparison** | **Time (min)** | **p-value** |
| --- | --- | --- |
| gallein vs vehicle | 30 | 0.0339 |
| gallein vs vehicle | 60 | 0.0006 |
| gallein vs vehicle | 90 | 0.0054 |
| gallein vs vehicle | 120 | 0.0234 |
